# Designing Tailored Web-Based Instruction to Improve Practicing Physicians' Preventive Practices

**DOI:** 10.2196/jmir.5.3.e20

**Published:** 2003-09-24

**Authors:** Linda L Casebeer, Sheryl M Strasser, Claire M Spettell, Terry C Wall, Norman Weissman, Midge N Ray, Jeroan J Allison

**Affiliations:** ^1^Division of Continuing Medical EducationUniversity of Alabama School of MedicineBirmingham ALUSA; ^2^Aetna Integrated Informatics, IncHartford CTUSA; ^3^University of Alabama School of MedicineDepartment of PediatricsBirmingham ALUSA; ^4^University of Alabama at BirminghamSchool of Health Related ProfessionsBirmingham ALUSA; ^5^University of Alabama School of MedicineDivision of General Internal MedicineBirmingham ALUSA

**Keywords:** Internet, World Wide Web, online, continuing medical education, continuing health care education, education theory, cognitive theory, Web site design, chlamydia screening

## Abstract

**Background:**

The World Wide Web has led to the rapid growth of medical information and continuing medical educational offerings. Ease of access and availability at any time are advantages of the World Wide Web. Existing physician-education sites have often been designed and developed without systematic application of evidence and cognitive-educational theories; little rigorous evaluation has been conducted to determine which design factors are most effective in facilitating improvements in physician performance and patient-health outcomes that might occur as a result of physician participation in Web-based education. Theory and evidence-based Web design principles include the use of: needs assessment, multimodal strategies, interactivity, clinical cases, tailoring, credible evidence-based content, audit and feedback, and patient-education materials. Ease of use and design to support the lowest common technology denominator are also important.

**Objective:**

Using these principles, design and develop a Web site including multimodal strategies for improving chlamydial-screening rates among primary care physicians.

**Methods:**

We used office-practice data in needs assessment and as an audit/feedback tool. In the intervention introduced in 4 phases over 11 months, we provided a series of interactive, tailored, case vignettes with feedback on peer answers. We included a quality-improvement toolbox including clinical practice guidelines and printable patient education materials.

**Results:**

In the formative evaluation of the first 2 chlamydia modules, data regarding the recruitment, enrollment, participation, and reminders have been examined. Preliminary evaluation data from a randomized, controlled trial has tested the effectiveness of this intervention in improving chlamydia screening rates with a significant increase in intervention physicians' chlamydia knowledge, attitude, and skills compared to those of a control group.

**Conclusions:**

The application of theory in the development and evaluation of a Web-based continuing medical education intervention offers valuable insight into World Wide Web technology's influence on physician performance and the quality of medical care.

## Introduction

The World Wide Web (Web) provides a delivery system for conveying complex, structured information to a large number of users without the barriers of time and geography [1]. Nearly all physicians have access to the Web, know how to use it, and access it for medical information [2]. Physicians reported most frequently using the Web for e-mail, medical information sources, travel information, product information, and professional association communications [3]. A particular patient problem was the most-common reason for seeking information through the Web. Credibility of the source, quick and 24-hour access to information, and ease of searching were most important to physicians [2]. Barriers to use included too much information to scan and too little specific information to respond to a defined question [2]. Many online medical resources lack design features that organize content and simplify usage; a dearth of well-designed applications in medical education has been noted [1].

In 2000, 96 continuing medical education sites were available; in 2001 this number had more than doubled to 209, with 18263 hours of continuing medical education credit offered online [4]. However, 28% of these sites contained only text. Only 17% of the sites were interactive, and 7% were guideline-based. Sklar noted that most online CME (Continuing Medical Education) offerings do not make use of unique computer capabilities to offer multiple pathways to learning and interactive responses [4]. Studies have shown that traditional CME lectures and simple information dissemination, similar to the text-only online sites, do not usually change physician practice patterns; although physicians may report that they intend to change their practice patterns after a traditional CME course, the evidence generally refutes this assertion [5- 7]. If the Web is to be used optimally as an intervention to improve physician performance and patient-health outcomes, physician interventions delivered through the Web must go beyond the simple posting of information. The design of these interventions will benefit from being informed by learning theories as well as the current evidence about which provider interventions are most effective in improving physician performance and patient-health outcomes.

### Learning Theories

Because of the potentials for high costs and adverse consequences of poor performance, medical education represents a major category of lifelong education [8]. Kearsley has catalogued over 50 major learning theories applicable to adult lifelong learning [9]. In choosing and applying learning theories to medical education over the physician's lifetime, key characteristics of the discipline determine which learning theories are most relevant. Cognitive processes including skills such as decision making, reasoning, and problem solving are critical in medical practice, leading to a focus on cognitive learning theories. Cognitive theories such as situated learning theory and cognitive flexibility theory are examples of cognitive theories particularly relevant to medicine [9- 11].

Situated learning, andragogy, and cognitive flexibility theories are examples of cognitive learning theories relevant to the design of physician education. Situated learning focuses on the social nature of cognition and the importance of authentic situations to learning; specifically this requires settings and applications to be relevant to the daily life of the learner [11]. Similarly, andragogy theory proposes that instruction should be task-oriented and presented in the relevant context of common tasks performed, should take into account the learner's knowledge and experience, and should be problem-centered not content-focused [12]. Cognitive flexibility theory emphasizes a case-study approach involving context-dependent and realistic situations. This theory addresses the nature of learning in complex domains and focuses on the transfer of knowledge and skills beyond the learning situation [10,13]. In general, cognitive frameworks suggest that the sequence and pace of instruction be controlled by the learner, and that the instruction be tailored or individualized to participant needs.

### Physician Change Theories

Several theorists have applied constructs specifically to the problem of how physicians in clinical practice learn and change.

First, motivation for physician learning has been linked to the nature of the problem, most frequently a specific patient problem as physicians seek information to deal with uncertainty in the clinical encounter: surprise, stress, and cognitive dissonance in this context may lead to information seeking [14].

Second, problem-based learning (PBL), a cognitive learning strategy, has been important in organizing undergraduate and graduate medical-education curricula but has not yet become the standard format for CME [15]. In problem-based learning, learners construct problem-oriented semantic networks (visually-depicted problem structures consisting of shapes or objects graphed to represent concepts with interconnecting lines indicative of relationships) that include cues from the context of professionally-relevant problems, fostering professional curiosity [16].

Third, application of a stages-of-change model to the question of how physicians learn and change has been suggested [17,18]. The stages of precontemplation, contemplation, preparation, action, and maintenance can be used to better understand a physician learner's development and readiness for learning [18].

Fourth, Schoen's model has described a process by which physicians reflect on their daily practice in order to continue to learn over time; practitioners engage in situated action until their expectations are not met and they experience a breakdown in their current work situation [19]. At that moment, practitioners stop and reflect, creating motivations to move beyond the breakdown through ongoing learning [19].

Finally, Fox and others have proposed that learning and change resulting from information-seeking behavior varies from stage to stage in a multistage process [20,21]. When information is sought that might indicate the need for complex changes in practice, other conditions of adoption must be met, including commitment to change, a conceptual basis for making the change, and time to deliberate over making the change. Information seeking might play various roles in this process accordingly [20].

### Appropriate Educational Delivery Systems

Not only is it important to examine which learning theories are the most applicable to medical education, but also which media or educational delivery systems are most supportive of relevant theories. Since situated learning focuses on the social nature of cognition and the importance of authentic situations to learning, computer-assisted and Web-based instruction are ideal formats for CME since they can tailor the learning process to the individual student by providing teaching and support in response to the individual's immediate needs. These forms of instruction can also bring experts to the learner, demonstrating clinical reasoning skills.

Learning through the Web can also be enhanced by immediate repetition, which helps the learner make new knowledge and skills explicit. These aspects put the student at the center of the learning processes, as suggested by situated learning theory, and make many resources available through a large number of different learning pathways and possibilities [22]. Computer-based models for problem-oriented learning and clinical reasoning are simpler and less expensive to produce than those models that depend on live interaction and can be extremely effective [22]. In addition to situated learning theory, cognitive flexibility theory also strongly supports the use of interactive technology and the use of clinical cases to emphasize knowledge construction rather than the transmission of information [13].

While not specifically related to medical education, a recent investigation of research involving the development of Web-based instruction was conducted to determine which instructional design models or approaches had been adopted for the design of Web-based instruction [23]. The majority of Web-based instruction was designed following existing instructional design models, primarily grounded in behavioral, not cognitive, learning theories [23]. The most-frequently used model in developing Web-based instruction was the standard Dick and Carey instructional model (assess, design, develop, implement, and evaluate) [24].Within this model, the most-frequently-used elements of the model were to: analyze learning contexts, learning tasks, and learners, as well as to determine delivery strategies, and to write and produce instruction. E-mail was the medium used most frequently for interaction. About half of those surveyed believed the existing instructional-design models were not appropriate for designing and developing Web-based instruction [24]. This information may help to explain data collected by Sklar in his examination of CME sites, in which he found that of the 96 CME sites available in February 2000, 28% contained text only, and 38% contained text and graphics. Only 17% of the sites were interactive, and 7% were guideline based. Sklar noted that most online CME offerings do not make use of unique computer capabilities to offer multiple pathways to learning and interactive responses [4].

In considering the appropriate theoretical context for the design of an intervention to improve physician performance in screening for chlamydia, we drew on the work of cognitive theorists, specifically work on the processes of assimilation and accommodation, and on stages of learner's development and readiness for learning. Because of its direct application to problem solving, we also used cognitive theoretical frameworks that suggest the learner control the sequence and pace of instruction, and that the instruction be individualized to participant needs.

### CME Interventions: Evidence of Effectiveness

As early as the 1970s, evidence suggested that traditional CME programming was not effective in facilitating changes in physician performance and changes in patient-health outcomes [25- 28]. Since that time, many well-designed research studies have only increased the evidence that traditional didactic teaching is not the most-effective method for influencing physician performance or patient-health outcomes [6,7,28- 32]. Many studies have demonstrated that CME conferences have little impact on improving professional practice or on improving patient-health outcomes. Didactic CME courses have also had weak effects on guideline adoption [5]. Interventions such as educational-outreach visits and patient-educational materials were more likely to improve physician performance and patient-health outcomes compared to single, episodic didactic sessions [5- 7].

Most recently, Davis and colleagues summarized the results of 14 randomized, controlled trials of physician education conducted between 1993 and January 1999, concluding, from this review at least, that didactic teaching sessions do not appear to be effective in changing physician performance [33]. There is evidence here that interactivity and sequencing of events (eg, 2 sessions held 1 month apart) increases learning effectiveness. Data from this study suggest that adding adequate needs assessments prior to the course, and/or adding enabling materials, such as patient-education materials or flow charts, to the material distributed during the course can improve course outcomes [33]. Peloso and Stakiw note that the ability to change practice is enhanced if the information presented is supported by published evidence, if the changes are endorsed by opinion leaders, and if there is opportunity for practice and feedback [34].

Across various reviews examining the effectiveness of interventions aimed at influencing physician behavior, the use of multiple interventions has been more successful than the use of a single episodic intervention [7,30,34- 36]. Hulscher and colleagues focused specifically on reviewing 55 studies involving more than 2000 health professionals and 99000 people; each of these trials tested interventions designed to improve prevention in primary care [37]. Evidence did not support the use of any specific strategy as the most-effective intervention to improve preventive practices in primary care. Reviewers concluded that tailoring interventions to address specific barriers to change in a particular setting is probably important, and that the effectiveness of multifaceted interventions over single interventions may be attributed, at least in part, to being able to address more barriers to change [37].

Others have used feedback to providers as an intervention to improve physician performance-with the assumption that knowledge of one's own performance, together with the ability to compare this performance against some reference level (internal or external) will facilitate improvement [38- 42]. In a review of 37 randomized, controlled trials of audit and feedback that included 4977 physicians, 28 studies measured physician performance, 1 study measured patient-health outcomes, and 8 studies measured both [37]. In 4 trials of audit and feedback versus no intervention, prescribing practices changed significantly. In 10 of 15 trials using audit and feedback plus educational materials or meetings versus no intervention, statistically-significant changes as a result of audit and feedback were demonstrated [37]. In 6 of 11 studies that included audit and feedback as part of a multifaceted intervention, there were significant improvements in physician performance; 1 of 2 studies measuring patient outcomes showed significant improvement in patient outcomes [37]. The results from the meta-analyses lead our research group to conclude that audit and feedback methods may contribute to change in physician behavior.

Based on a MEDLINE literature search from June 2000 through June 2002, there have not yet been enough rigorous trials to determine the effectiveness of Web-based courses in improving physician performance and patient-health outcomes, therefore, meta-analyses have not been conducted. However, from the evidence of effectiveness in other reviews [5,6,27- 37,43] we have drawn the following design principles for our chlamydia Web-based intervention.

### Design Principles

We abstracted the following Web-design principles for physician-education Web sites from learning and change theories, as well as from the evidence of what works in continuing medical education:

- Office-practice data as needs assessment
- Multimodal strategies
- A series of modules rather than one single, episodic educational event
- Contextual learning in the form of clinical cases
- Tailoring based on individual responses
- Interactivity
- Audit and feedback
- Evidence-based content
- Credibility of the organization providing the Web site and instruction and of any agency providing grant support for the education or site
- Patient-education materials
- Ease of use of the site and ease of navigation
- Design for the "lowest technological denominator" in hardware.

### Evaluation of the Effectiveness of Online Courses

Although the Cochrane Collaborative has produced overall reviews of the effectiveness of CME interventions, audit and feedback, live conferences, and academic detailing, there has been too little overall evaluation using randomized, controlled trials of online-course interventions to produce a review of their effectiveness. Individual studies have tended to be descriptive and have focused on participant reactions rather than improvements in physician performance and changes in patient-health outcomes. For example, a descriptive study of online interactive pathology case studies details the interactive format of CME cases that allow participants to submit immediate comments or criticism to case authors and to receive immediate feedback on their own performance; these features are normally unavailable in traditional CME courses. The evaluators note that the dynamic environment of the Web allows development of flexible forms of CME for the physician [44].

A descriptive evaluation of a hybrid delivery system was conducted [45]. The system merged Web documents, multimedia including CD-ROMs, and asynchronous learning communications to enable self-paced instruction and collaborative learning. The course was effective in increasing knowledge ( *P*= .05) and in improving self-reported competency ( *P*= .05) in dermatologic office procedures. Participant satisfaction was high with self-paced instruction as well as with sharing information with colleagues.

A Web-based tutorial system was compared to a print tutorial system with residents for the management of care following acute myocardial infarction. Immediate post-test scores were similar in both groups, but Web users spent less time studying, producing greater learning efficiency ( *P*= .04). Web users were more satisfied with the learning experience ( *P*= .001). Knowledge decreased to the same extent in both groups at 4 to 6 months following the instruction. Authors commented that further research is needed to identify instructional features that motivate greater final learning and retention [46].

Examining the same Web-based system used with residents for the management of care following acute myocardial infarction, on average, users accessed less than half of the guideline passages and little of the graphic evidence. Greater use of guideline passages was correlated with greater immediate learning, but use of graphic evidence was not. Authors commented that further research is needed to integrate clinical-trial evidence with guideline-based education [46].

A pilot evaluation of a Web-based curriculum reviewed occupational and environmental health or medicine components of 2 undergraduate degree courses and 2 postgraduate courses including interactive components; 12 students achieved the main learning objectives. Participants valued the flexibility, timeliness, efficiency, and breadth of access to relevant information offered by the Web [47].

Most Web-based materials have not been subjected to external assessment for quality. An online questionnaire was developed covering general suitability, local suitability, user interface, educational style, and a general review-and was piloted in 3 subject areas: general chemistry, radiology, and medical physics, focusing on undergraduate teaching. The evaluation methodology was found to work well for highly-structured and formal content and may have value in helping those developing undergraduate curricula to identify appropriate Web-based materials for integration into the curricula [48].

While these studies are useful as formative evaluations of Web courses, rigorous summative evaluations are needed. Ward notes that the Web provides powerful tools for learning medical education and will alter how the discipline is taught; for the drive to incorporate such technologies threatens to outstrip an overall understanding of how they can be used most effectively [22]. To avoid this, educational design must be sound and evaluation including cost-effectiveness must be rigorous [22].

## Methods

### Chlamydia Screening Web-Based Intervention Design

In collaboration with a large national health maintenance organization, a needs assessment of current practice patterns related to screening for chlamydia was conducted on a national sample of physicians from 16 states. A series of three 1-hour Web modules was designed to increase primary-care physician screening rates in the population of women ages 16 to 26 years. A physician was identified as eligible for participation if the physician's office participated in a specific health plan, if the physician's office had at least 10 sexually-active patients between the ages of 16 and 26 years, and if the physician had Internet and e-mail access. Physicians were recruited to participate by fax and FedEx communications. Physicians were notified of the online availability of each module by e-mail announcements as well as a series of e-mail reminders, all containing the link to the modules within the e-mail messages. The program invitation was received by 3067 physicians. Upon complete login, physicians were randomly assigned to either the intervention arm or the control arm of the study.

### Adequate Needs Assessment

Baseline data demonstrated an average chlamydial screening rate of less than 20%, with the lowest rates in the group of sexually-active young women ages 21 to 26 years and higher rates in the 16 to 20 year-old group. Screening rates and the process used to abstract them from administrative data have previously been reviewed (Ray M, et al. Unpublished data, 2001).

### Multimodal Intervention

Based on the evidence that single CME events are unlikely to improve physician performance and enhance patient-health outcomes, and evidence of the "decay effect" (a reduction of effect over time) of interventions, we used a series of 3 modules rather than 1. To enhance our theoretical chances of effect, each module would use a multimodal approach. Each module included the following components:

- Today's cases: vignette series of primary care cases of young women 16-26 years of age
- My screening data: audit and feedback at the office level of chlamydia screening data
- Help for my office: patient brochures and physician screening guidelines.

**Figure 1:**
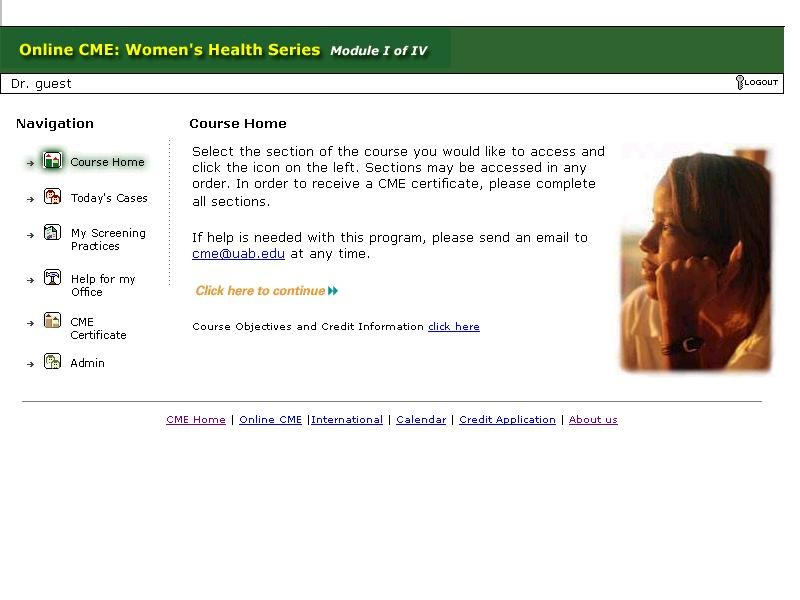
Chlamydia module I course home page

Figure 1 displays the course home page of the first module of the chlamydia intervention.

### Problem-Based Learning

The section titled "Today's cases" was developed based: (1) on studies demonstrating that physician information seeking on the Web is most-frequently connected to searching for information related to a specific patient problem and (2) on adult learning principles that focus on the relevance of instruction to an adult's work and life. Theoretical constructs from problem-based learning and from situated learning theory are central to this design in creating an authentic contextual process through the use of cases and in putting the learner at the center of the learning processes. The Schoen cyclical model of action, breakdown, and reflection was selected as the central focus of the chlamydia intervention. This parallels a physician's motivation to seek information for purposes of patient problem-solving [19]. Each module includes one unfolding case, as well as a series of brief vignettes. The unfolding case at the beginning of each module allows physicians to obtain information on the patient's history, physical exam, and tests; learners are then asked questions concerning diagnosis and management of the presented patient.

### Tailored Interactive Responses

More prominently featured in earlier forms of behavioral theory-based computer-assisted instruction, tailoring and branching of responses to meet individual needs has not been broadly applied to the development of Web-based instruction. Modules were developed with individualized responses to multiple case-based and practice-based questions, leading to over 300 possible permutations of pathways throughout individualized modules. Using methods reported by Kreuter et al [49], tailoring of responses is transparent to the user and occurs in real time as the physician accesses the program.

In addition to physician case responses as a means of tailoring and remediation, we used the theoretical constructs related to stages of change to develop tailored pathways throughout the modules. A series of attributes were attributed to various stages of change. For example, if a learner considered the overall prevalence of chlamydia to be less than 1% or if the learner did not take sexual histories, he or she would be categorized as precontemplative and branched to pathways designed to heighten awareness of the growing prevalence of chlamydia or the importance of taking a sexual history. If, however, the learner demonstrated skill in taking a sexual history and reported updating sexual histories at each visit with young female patients, the learner would be branched to pathways designed to reinforce current skills and evidence that supports the need to continue to focus on these activities [49].

Dick and Carey have noted the frequent lack of emphasis in instructional design on practice and rehearsal of skills, resulting in a lack of transference of skills into practice [23]. Using the reflection on practice described by Anderson et al [50], participant responses to case questions in the chlamydia intervention, are graphed-so the learner can compare his or her response to that of other participants-and responses are followed by expert comment from the faculty based on current evidence and guidelines.

### Audit and Feedback

**Figure 2:**
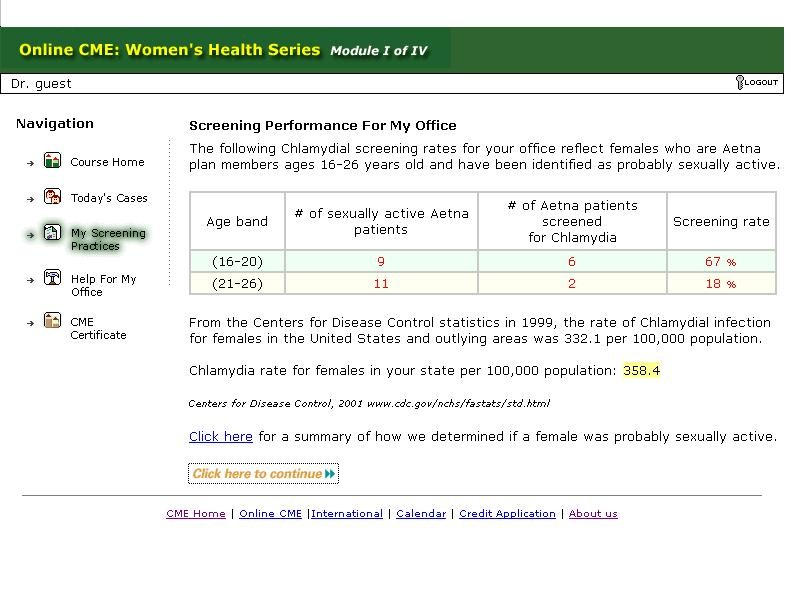
Chlamydia module I "screening performance for my office" page

The collaboration of a university medical center with a large national health maintenance organization has significantly enhanced the development of this Web-based intervention in several ways. First, administrative data provided a solid basis for needs assessment, as well as baseline measurement of physician performance in chlamydia screening, by identifying sexually-active women between the ages of 16 and 26 years. Second, a list of physicians was linked to patients as the primary care providers for these patients for the purposes of intervention. Administrative data can then be used to provide data, following the intervention, on its effectiveness. For the purposes of the intervention itself, data at the individual office level, as well as the regional level, could be provided to participants in the Web intervention. Using a Structured Query Language (SQL) database server and participant log-on criteria, each participant was able to access office-level data as a means of audit and feedback. A depiction of "Screening performance for my office" is in Figure 2.

Initial data demonstrated that this page was the most-frequently visited page. In the evaluation survey, this module element was cited as the most useful.

### Supporting Published Evidence

Core messages were developed based on a new set of guidelines developed by the US Preventive Task Force [51], as well as focus groups with clinical experts. Focus group discussions included a pediatrician, a general internist, and 2 infectious disease specialists. Three main points were stressed in each of the 3 modules:

- Sexually-active women between the ages of 16 and 26 years are at highest risk for chlamydial infections that can lead to pelvic inflammatory disease, increased risk of HIV infection, and infertility.
- New urine-based testing allows screening for chlamydial infection without a pelvic examination.
- Chlamydial infections can be effectively treated with a 1-dose antibiotic, increasing the likelihood of adherence to treatment [51].

Case answers were supported by evidence from the literature and were referenced to provide physicians with additional sources of information.

### Enabling Materials

In the section labeled "Help for my office" the following materials have been included to support the physician in translating new knowledge and skills into the practice environment:

- Patient education brochures on sexually transmitted diseases (STDs) and chlamydia, including Spanish and English versions
- Brief summary of physician guidelines
- Access to full text of new screening guidelines
- Confidentiality policy regarding disclosure of teen information to parents.

## Results

### Preliminary Evaluation Data

In the formative evaluation of the first 2 chlamydia modules, data regarding the recruitment, enrollment, participation, and reminders have been examined. From the initial recruitment period (February 1, 2002, through October 2, 2002) there were 3067 primary care physicians in 1045 offices that received fax materials recruiting their participation. Of those, 463 physicians registered by fax for the course and provided their e-mail addresses. Although 210 physicians logged on, only 180 physicians completed the first module. Among the physicians completing the module, 92 were randomly assigned at the time of log on to the intervention group and 89 to the control group. The second module was completed by 134 physicians. For the third and forth modules, 96 and 61 physicians participated, respectively.

Physicians were asked to assess the usefulness of particular course elements: cases, screening information, "My screening practices," and "Help for my office." Table 1 displays physician perceptions of usefulness corresponding to the aforementioned course elements included in the first 3 modules. Cases and screening information were found to be most useful across the first 3 modules.

**Table 1:** Intervention physician perceptions of "usefulness" of course elements

**Usefulness of:**	**Module I**	**Module II**	**Module III**	**Module IV**
Cases	41	31	31	NA*
Screening information	41	22	7	NA
My screening practices	5	7	3	NA
Help for my office	2	3	0	NA

^*^ NA = Not Available

Chlamydia knowledge, attitudes, and skills of intervention physicians and control physicians were measured and compared following the intervention. A 21-item post-test was conducted following participation in the fourth module. The mean number of correct responses was calculated for each group. The mean number of correct answers to the content questions collected from module IV demonstrates a significant difference between physicians in the intervention and control groups. The control-group mean score of correct answers was 78.3 compared to 93.0 for the intervention group. A paired t test calculation determined the difference to be statistically significant at an alpha level of .0003.

We are conducting a further evaluation of the chlamydia screening Web-based intervention design. Additional outcomes are the differential improvement in screening rates of the 2 study arms as ascertained from administrative data. Patient-level multivariable analyses will adjust for the extrabinomial variation resulting from patients being nested within physician offices from the group randomized design. Screening rates from the calendar year of 2001 will be compared with those from 2002 to determine both within-group and between-group differences.

Analyses are scheduled to be complete by the end of 2003. Two other evaluation studies of this intervention have been initiated; the intervention is being adapted to a project designed to improve the diagnosis and management of glucocorticoid-induced osteoporosis and a second project with the goal of increasing the adoption of multiple secondary prevention guidelines for the post myocardial infarction patient.

## Discussion

While traditional instructional design models have been applied to the creation and development of Web activities, the failure to apply cognitive learning theory to Web design creates a challenge for those developing Web-based instruction for physicians. The need for rigorous evaluation of Web-based interventions has been well documented but has yet to produce enough evidence to create a performance benchmark. Application of theory and rigorous evaluation will be crucial over the next decade to those who wish to use the Web to influence physician performance and the quality of medical care.
